# The Influence of Individual and Situational Factors on Teachers’ Justice Ratings of Classroom Interactions

**DOI:** 10.3389/fpsyg.2022.789110

**Published:** 2022-06-14

**Authors:** Scarlett Kobs, Antje Ehlert, Jenny Lenkeit, Anne Hartmann, Nadine Spörer, Michel Knigge

**Affiliations:** ^1^Division of Rehabilitation Psychology, Department of Rehabilitation Sciences, Humboldt University, Berlin, Germany; ^2^Special Educational Needs With Focus Learning, Structural Unit Educational Science, University of Potsdam, Potsdam, Germany; ^3^Psychological Primary School Pedagogy, Structural Unit Educational Science, University of Potsdam, Potsdam, Germany

**Keywords:** classroom interactions, justice, special educational need, justice ratings, inclusion

## Abstract

Teachers, as role models, are crucial in promoting inclusion in society through their actions. Being perceived as fair by their students is linked to students’ feelings of belonging in school. In addition, their decisions of resource allocations also affect students’ academic success. Both aspects underpin the importance of teachers’ views on justice. This article aims to investigate what teachers consider to be just and how teacher characteristics and situational factors affect justice ratings of hypothetical student-teacher-interactions. In an experimental design, we randomly varied the description of the interacting student in text vignettes regarding his/her special educational need (SEN) (situational factor). We also collected data on teachers’ attitudes toward inclusion and experiences with persons with disabilities (individual factors). A sample of in-service teachers in Germany (*N* = 2,254) rated randomized versions of two text vignettes. To also consider the effect of professional status, a sample of pre-service teachers (*N* = 275) did the same. Linear mixed effect models point to a negative effect of the SEN on justice ratings, meaning situations in which the interacting student is described with a SEN were rated less just compared to the control condition. As the interacting student in the situations was treated worse than the rest, this was indicative for the application of the need principle. Teachers with more positive attitudes toward inclusion rated the vignettes as significantly less just. Professional status also had a negative effect on justice ratings, with in-service teachers rating the interactions significantly lower than the pre-service teachers. Our results suggest that the teachers applied the principle of need in their ratings. Implications for inclusive teaching practices and future research are discussed further.

## Introduction

Providing sufficient education for all children in mainstream schools has become an important agenda of recent education reforms around the world (The United Nations, 2006; Ainscow, 2020). Understanding inclusive education as shared “values of respect for difference and a commitment to offering all students access to learning opportunities” (Ainscow, 2020, p. 12), issues of justice are simultaneously raised here as well. Thus, a broader understanding of inclusion that recognizes a wide variety of individual needs beyond disability is adapted here. With the ratification of the UN Convention on the Rights of Persons with Disabilities (The United Nations, 2006) in Germany in 2009, students with diverse backgrounds and differing educational needs are increasingly taught in the same classroom. Students with varying needs according to their socio-emotional development as well as students with differing approaches to learning are more common in everyday learning settings in Germany (Sekretariat der Ständigen Konferenz der Kultusminister der Länder, 2022). This ongoing reform has sparked a discourse around the “correct” distribution of resources in favor of inclusive education, and ongoing reflections on educational justice extended to yet another group of students (Werning, 2014; Ainscow, 2020). How (in)justice and inclusion relate to each other in a school context is a question that has been discussed from different perspectives (e.g., specifically for the German education system; see Kiel and Kahlert, 2017). Beside ethical and normative views on this question, regarding a successful implementation of inclusive education in public institutions like schools, it is crucial to understand how perceptions of (in)justice relate to traditional and rather new – inclusive – practices.

The everyday experiences of students in school shape their understanding of our society’s values (Gorard, 2011; Resh and Sabbagh, 2014). Having “fair” teachers is an important aspect of this. Interactions with teachers as a source of justice experiences in school are linked to a student’s sense of belonging, which is particularly important in inclusive learning environments, student’s motivation (Donat et al., 2016; Umlauft and Dalbert, 2017), and their academic achievement (Dalbert, 2011). The teachers’ key role in educating our future citizens places a heightened importance on their actions as they represent and impart our society’s values to the students. Accordingly, what teachers believe to be just or unjust in inclusive teaching settings is highly relevant as previous research points out a strong connection between hypothetical and real actions (Eifler, 2008).

Deviations from an equal distribution of attention and appreciation in favor of a needs-based distribution can be important aspects of inclusive teaching settings (Ainscow, 2020). How teachers evaluate such interactions, dependent on noticing and interpreting special needs of students, is essential. Individual factors of the teacher, like his/her attitudes toward inclusion and experiences with persons with disabilities, might also affect their justice ratings. In this study, the focus is on justice ratings of hypothetical classroom interactions in inclusive settings by teachers, specifically interactions between teacher and students. We investigate situational as well as individual factors and their link to justice ratings of these situations. By experimentally varying the situational information given about the student and reducing complexity by using text vignettes, we gain insight into the teachers’ justice cognitions. In doing so, we can examine the causal effects of special educational needs (SEN) of students in inclusive learning settings on the justice ratings of teachers. General principles derived from these ratings can contribute to the existing discourse on justice in inclusive education settings.

## Justice in Psychological Research

Referring to research in the field of social psychology, the term “justice” is defined as individually experienced justice of social interactions (Mikula, 2002; Gollwitzer and van Prooijen, 2016). In the literature, there is disagreement about the extent to which justice might be an aspect of morality, and morality is seen as one of many motives for justice. It seems to be established that morality and justice are linked yet distinct constructs (Skitka et al., 2016). The focus of this study is to investigate what is personally considered as just or unjust (Peter et al., 2013; Gollwitzer and van Prooijen, 2016). When assessing (in)justice there are four dimensions usually distinguished: distributive, procedural, interpersonal, and informational justice (Colquitt and Greenberg, 2003).

*Distributive justice* is defined as the perceived justice of decision and/or allocation outcomes (Colquitt and Greenberg, 2003; Gollwitzer and van Prooijen, 2016). A scarce resource in school might be the attention and time a teacher can spend with a student. Previous research has established that there are three different principles usually applied when people assess justice in this area: the *principle of equality*, the *need principle*, and the *principle of effort* (Mikula, 2002; Berti et al., 2010; Peter et al., 2013). Allocating resources based on the *principle of equality* results in the same outcome for each recipient regardless of their individual effort or needs. The *need principle* states that students get resources based on their individual needs. Lastly, following the *principle of effort* the resource distribution is based on individual input or achievement. For example, students who are doing well in class would get more attention from their teacher (Mikula, 2002; Gollwitzer and van Prooijen, 2016; Ehrhardt-Madapathi et al., 2018). The second dimension of justice is *procedural justice*. It is concerned with the perceived justice of the process that led to a decision or allocation of outcomes (Colquitt and Greenberg, 2003; Gollwitzer and van Prooijen, 2016). *Interpersonal* and *informational justice* are concerned with communication and interactions. *Interpersonal justice* refers to one’s perception of interaction and communication. It encompasses respectful and appropriate interpersonal interactions (Colquitt and Greenberg, 2003; Peter et al., 2013; Fischer, 2016; Ehrhardt-Madapathi et al., 2018). *Informational justice* refers to adequate explanations and transparency, especially in communicating a decision (Colquitt and Greenberg, 2003; Peter et al., 2013).

The present article will focus on distributive and interpersonal justice for three reasons. First, in the UN Convention on the Rights of Persons with Disabilities (The United Nations, 2006), it is proposed that the diversity of every learner should be respected and educational institutions should enable every member of society to participate in the very same. These claims are reflected in both justice dimensions. Second, previous research suggests that interactions concerning distributive and interpersonal justice can easily be observed in class (Ehrhardt-Madapathi et al., 2018). Third, when asked how an ideal or just teacher would behave students usually describe behaviors touching on issues of distributive as well as interpersonal justice (Berti et al., 2010; Gorard, 2012; Peter et al., 2013). Regarding distributive justice, students express conflicting ideals representing the three justice principles. For instance, students wish to be praised when deserved, but also disapprove of favoring hard-working students (Ulich, 2001; Gorard, 2012). The application of the principle of equality and the effort principle are in conflict here. In addition, students wish everyone to be treated the same while also expressing that students in need should get additional help (Ulich, 2001; Berti et al., 2010; Gorard, 2012; Peter et al., 2013). This points to conflicting ideals regarding the principle of equality and the need principle. In contrast, teachers of an Italian study seemed to prefer the principles of need and effort when distributing goods and resources in the classroom (Berti et al., 2010). On the other hand, Iranian English language teachers reported a preference for the principle of equality when distributing resources (Estaji and Zhaleh, 2021). In terms of interpersonal justice, students wish to be treated with respect and not be humiliated in front of their class (Ulich, 2001; Gorard, 2012). Likewise, teachers also consider treating students with respect as an important factor when communicating in class (Berti et al., 2010).

### Research on Justice in School

Justice experiences in school can be investigated from various perspectives. As stated above, students’ experiences of justice in school are of particular importance since these experiences shape their ideas of what justice means in our society (Gorard, 2011, 2012). Teachers and teacher actions often represent the rules and values of their school and by that, they shape students’ experiences of their daily school life as well as their representations of our society’s values (Gorard, 2011). What teachers believe to be just or unjust is highly relevant since this can be assumed to translate into actions (Eifler, 2008; König et al., 2014). For instance, Ehrhardt-Madapathi et al. (2018) found a bidirectional relationship between teachers’ sense of justice regarding their own actions and students’ behavioral problems. Students reacted to unfair treatment with behavioral problems which led to a further decrease in teacher sense of justice, “creating a vicious circle” (Ehrhardt-Madapathi et al., 2018, p. 359).

One way to learn more about teacher justice in school is to ask students how they perceive their teachers. In several studies, students reported their daily interactions with their teachers to generally be unjust (Abs et al., 2007; Berti et al., 2010; Gorard, 2011). On the other hand, there is also evidence that students perceive their teachers’ actions as generally just (Donat et al., 2016, 2018a,2018b). In an observation study conducted in 45 primary and secondary schools in Germany, Prengel (2019) found overall positive interactions between teachers and students, further supporting the evidence of generally just teachers.

So far, little is known about teachers’ justice experiences in school. The few studies investigating this area present inconsistent findings. Evaluating a democracy project, Abs et al. (2007) found that teachers rated their own actions toward students as “sometimes” to “rarely” unjust. However, asked about students’ actions toward them, teachers rated them more often to be unjust (Abs et al., 2007). In contrast, in a study by Ehrhardt et al. (2016) teachers rated their actions concerning the allocation of praise and attention to be very just while also reporting that they were aware of the sensitivity of these allocations (Ehrhardt et al., 2016). The comparability of these studies is limited as Abs et al. (2007) did not instruct the teachers to assess specific actions. How these perceptions relate to students’ (learning) outcomes has yet to be investigated. The studies described so far have in common that participants or observers were asked to assess the behavior or interactions of a person in specific situations or toward them in terms of justice. In questionnaire studies, the instructions for students and teachers usually do not specify which student’s or teacher’s actions should be assessed. This can cause respondents to refer to a “mean” of student’s/teacher’s behavior which could limit the significance of the reported justice experiences (Molinari et al., 2013). In addition, recent experiences with one person or experiences that elicited very strong emotional reactions could overshadow other more positive interactions and thus create a bias in the reports of the participants. One way to avoid these problems is the use of hypothetical descriptions of a teacher’s behavior.

In a study with pre-service teachers, Kobs et al. (2021) used hypothetical descriptions of student-teacher-interactions to investigate the influence of information about a special need of the interacting student on justice judgments. They found that pre-service teachers rated the situations less just if the interacting student was described with having behavioral difficulties compared to the same situation with a neutral description of the student. The same pattern was found for situations focusing on the distribution of teacher resources when the student was described with learning difficulties. Kobs et al. (2021) concluded that prospective teachers evaluated distributive student–teacher-interactions in line with the principle of need. This was not entirely the case for interactions with a focus on interpersonal justice. However, the transferability of these results is limited since the sample consisted of pre-service teachers who generally did not have a lot of experience with teaching in a heterogeneous classroom.

As described above, students and teachers sometimes have contradictory ideals when it comes to justice in the classroom. However, both groups emphasize fairness as an important characteristic of a “good” teacher (Wilbert and Gerdes, 2007). Overall, these studies highlight the importance of and need for further research on the topic of justice in school.

### Potential Factors Influencing Justice Ratings of Classroom Interactions

Previous research has established that the assessment of a hypothetical behavior and a participants own behavior can be linked (Eifler, 2008; König et al., 2014). Investigating how teachers rate justice of hypothetical classroom interactions could provide insight into what teachers believe to be just interactions. Therefore, it is essential to investigate factors potentially influencing these justice ratings. It is important to differentiate between situational and individual factors. Individual characteristics of the participants evaluating interactions can influence their perception as well as situational factors, which they might not be able to control (modeled here via experimental variations). Thus, we choose to consider both individual and situational factors.

#### Student Characteristics

Student characteristics are important situational factors impacting teaching in diverse classrooms. Considering the focus on distributive and interpersonal justice, the following issue might arise: Teaching children without and with a SEN in one classroom can evoke conflicting approaches to teaching – supporting children in need individually or giving them additional attention (principle of need), and equally distributing the teacher’s attention among all students (principle of equality). In the presence of a SEN, treating each student the same, regardless of their individual needs, may be judged to be less fair than without a SEN present. Furthermore, if we consider inclusive education as recognizing the individual needs of every student, fostering learning environments correspondingly and enabling them to actively participate in our society (Piezunka et al., 2017; Ainscow, 2020), parallels to the above-mentioned definitions of the two justice dimensions arise. This concept of inclusive education reflects respectful and dignified interpersonal interactions in the sense of interpersonal justice. However, pre-service and in-service teachers in various studies have expressed concerns about teaching children with certain SEN in mainstream classrooms (Avramidis and Norwich, 2002; Ruberg and Porsch, 2017). These concerns may affect the justice ratings of the described interactions with and without information about a child’s SEN.

These concerns may be fueled by stereotypes or beliefs the (pre-service) teachers hold. In that sense, their beliefs about specific student characteristics might affect how they assess classroom interactions or other aspects of teaching (Hirschauer and Kullmann, 2010). Focusing on student’s ethnicity and social background, Lorenz et al. (2016) conducted a longitudinal survey among primary school teachers. They found a negative bias of the teachers toward students of Turkish origin, and from a lower social background, expecting them to achieve less than students of other groups during the school year (Lorenz et al., 2016). In a similar approach, teachers in preparatory service were asked to grade fictional students in a classroom in mathematics (Kaiser et al., 2015). Among other aspects, information on the social background (cultural capital) of the students was randomly varied. Kaiser et al. (2015) found no influence of cultural capital on the grade given. Both studies report interesting findings about stereotypes and teacher assessments, however connections to justice in school were not investigated. As described above, Kobs et al. (2021) investigated the influence of SEN on justice ratings among student teachers and found that equal treatment was considered less just if the interacting student was described with behavioral problems. This was also true for situations illustrating issues of distributive justice and a student with learning difficulties (Kobs et al., 2021). Whether these findings apply to in-service teachers as well is unknown. So far, similar investigations focusing on student characteristics and their link to justice ratings in inclusive teaching settings have not been conducted. Kobs et al. (2021) supposed that individual characteristics may also affect justice ratings. This is further investigated in the following sections of this paper.

#### Teacher’s Attitudes Toward Inclusion

It can be assumed that individual factors are of great importance when investigating justice ratings. By focusing on interactions in inclusive classrooms, a teacher’s attitudes toward inclusion might be an important aspect to consider. Given the established positive relationship between knowledge about SEN or inclusion in general and attitudes toward inclusion (Avramidis and Norwich, 2002; de Boer et al., 2011), more positive attitudes toward inclusion might lead to a heightened awareness for the individual needs of a student. This in turn, could affect justice ratings, specifically an application of the principle of need in the formation of justice ratings. However, a number of studies report (pre-service) teachers’ mostly negative attitudes toward teaching students with behavioral problems as well as learning difficulties in mainstream classrooms (Avramidis et al., 2000; Avramidis and Norwich, 2002; de Boer et al., 2011; Lübke et al., 2016; Ruberg and Porsch, 2017). Children with either one of these SEN are commonly taught in mainstream schools in Germany (Statistisches Bundesamt, 2017; Sekretariat der Ständigen Konferenz der Kultusminister der Länder, 2021). Considering these factors, it is relevant to investigate the effects of these specific SEN on the justice ratings of in-service teachers. It is also relevant here to see, whether attitudes toward inclusion generally affect justice ratings in the described way.

#### Experiences With Persons With Disabilities

Given the inclusive setting of our vignettes, experiences with persons with disabilities may be an essential factor when investigating justice ratings. Those experiences have been investigated to a great extent in regard to their influence on teachers’ attitudes toward inclusion (Avramidis et al., 2000; Avramidis and Norwich, 2002; Forlin et al., 2010; de Boer et al., 2011; Kim, 2011; Bosse and Spörer, 2014; Hellmich and Görel, 2014; Ruberg and Porsch, 2017). The so called “contact hypothesis” suggests that teaching children with special needs in their classroom leads to a positive change in teachers’ attitudes. Avramidis and Norwich (2002) literature review on the topic reported mixed results about this hypothesis. However, a more recent review by de Boer et al. (2011) supports the “contact hypothesis.” Applying this hypothesis to justice, more contact to students or family members, friends, etc. with SEN could also be linked to teachers being more sensitive to justice-relevant situations and individual needs of students. Since inclusion has been in practice longer in primary schools than in secondary schools in Germany the “contact hypothesis” and its transfer to issues of justice could be relevant here as well. Again, justice ratings in line with the need principle might be expected of teachers with more experience (personal or professional) with persons with disabilities.

#### Teaching Experience

Teaching experience may also be connected to teachers’ justice ratings of inclusive classroom situations. Its association with attitudes toward inclusion has been investigated in previous research. Studies show a negative link between increasing teaching experience and attitudes toward inclusion (Avramidis and Norwich, 2002; de Boer et al., 2011; Saloviita, 2020). Younger teachers with less experience seem to have more positive attitudes toward inclusion (Avramidis and Norwich, 2002; de Boer et al., 2011). Having more than 10 years of teaching experience may be a turning point, since teachers with more than 10 years of teaching experience seem to be more reluctant toward inclusion (Avramidis and Norwich, 2002; de Boer et al., 2011). However, Avramidis and Norwich (2002) also report that several studies did not find a connection between years of teaching experience and attitudes toward inclusion. If these findings on attitudes toward inclusion can be adapted to justice ratings of classroom interactions, less critical justice ratings of situations with a child with SEN present could be expected from more experienced teachers.

It is also important to look at teaching experience and its potential link to justice in terms of professional development. Following a competency-based approach, pedagogical knowledge is a central competence of a teacher (Shulman, 1987; Shavelson, 2010; Kunter, 2013). Knowledge about students’ specific needs that enable them to learn are highly relevant in diverse classrooms (König et al., 2014; Voss et al., 2015). Several studies suggest a positive link between pedagogical knowledge and practical experience (König and Klemenz, 2015; Voss et al., 2015; Mertens and Gräsel, 2018). Noticing and interpreting classroom situations is another important aspect of professional competence. It is often referred to as a teacher’s situational cognition and fosters a teacher’s ability to deal with heterogeneous learning groups in the classroom (Seidel and Prenzel, 2008; König et al., 2014). Research has shown that expert teachers identify relevant instructional situations more precisely and correctly than novices (König et al., 2014). They also interpret classroom situations more quickly than novices (Seidel and Prenzel, 2008; König et al., 2014; Paseka and Hinzke, 2014). In-service teachers should therefore be better at interpreting the situational factor SEN than pre-service teachers. They should in turn, by applying their theoretical and practical knowledge about SEN and therefore referring to the principle of need, rate the described situations less just than pre-service teachers.

### Research Questions and Hypotheses

There remain several aspects about justice cognitions in school that have yet to be investigated. Kobs et al. (2021) found a significant link between the context factor “SEN of a student” and pre-service teachers’ justice ratings of hypothetical text vignettes that describe inclusive teaching interactions. Whether this can be applied to in-service teachers is unknown. A link between a teacher’s attitudes toward inclusion and justice ratings of inclusive teaching settings stands to reason, however, it has not been investigated so far. As with the situational factor, none of the above-mentioned individual factors have so far been investigated in their relation to subjective justice ratings. This results in the following four research questions:

(1) In what way does information on a child’s SEN affect in-service teachers’ justice ratings of inclusive teaching situations?
(2) How can teachers’ attitudes toward inclusion in school be linked to justice ratings of inclusive teaching situations?
(3) Do experiences with persons with disabilities influence in-service teachers’ justice ratings?
(4) Is there a link between teaching experience and justice ratings of inclusive teaching practices?

Following these research questions and under application of the described research, the following corresponding hypotheses are derived:

(1) Following the principle of equality, equal treatment is rated as fair without further context information on the interacting student. With additional information about a SEN of the student, equal treatment is considered to be less just under the principle of need.
(2) Positive attitudes toward inclusion could be linked to a greater awareness for the specific needs of children with SEN and thus lead to a lower justice rating of equal treatment in accordance with the principle of need.
(3) Personal and professional experiences with persons with disabilities could raise awareness to the individual needs of children with SEN in the classroom. Therefore, we hypothesize that teachers with more personal and/or professional experience with persons with disabilities rate the described interactions as less just if a child with a SEN is described, again in line with the need principle.
(4) Since practicing teachers have more knowledge about and experience in teaching in heterogeneous classrooms than prospective teachers, we hypothesize that practicing teachers apply the principle of need and assess the described student–teacher-interactions less just than pre-service teachers if the student is described with SEN. Based on research about attitudes toward inclusion and teaching experience, we expect practicing teachers with less teaching experience to rate the student–teacher-interactions less just than teachers with more teaching experience.

## Materials and Methods

### Sample

The study is composed of two samples. The first sample was obtained within the framework of the project “Evaluation of inclusive schools in the state of Brandenburg, Germany^1^.” Data of 2,305 in-service teachers (84% female, 0.05% diverse) from the federal state of Brandenburg in Germany were collected. The participants were on average 49 years old (*SD* = 10.36). Most of the educators mainly taught in primary schools (64%), around a third of the sample said they mainly taught secondary students (31%) and the remaining teachers taught students of all age groups (5%). The survey was conducted in January and February of 2019. Data of 39 participants were excluded due to missing values on control or independent variables. Further, 12 participants who identified as “diverse” were excluded from analyses due to their very small proportion in the sample. Hence, our analyses included data of 2,254 participants.

To investigate research question 4, the second sample was composed of 284 pre-service teachers (51% female, 0.007% diverse, 14% not specified) studying to become secondary teachers at the University of Potsdam in the state of Brandenburg. Data was gathered as part of the project “Professionalization of (prospective) teachers in the field of inclusion”^2^ (Knigge et al., 2020). At the time of the survey, the pre-service teachers were on average in their 4th Bachelor’s semester (*SD* = 2.14) and were about 23 years old (*SD* = 4.63). The survey was carried out in the winter term of 2017/18. Data of nine students were excluded because of missing values on the dependent variable.

### Instruments

We used text vignettes to systematically investigate the influence of the context factor SEN on justice ratings of teachers – a common method in justice research (Atria et al., 2006; Steiner and Atzmüller, 2006; Auspurg et al., 2009; Weibler and Feldmann, 2012; Liebig et al., 2015). The situational descriptions are based on a psychological understanding of justice and represent student-teacher interactions in class [see Kobs et al. (2021) for details on the development of the vignettes]. A focus on two of the four justice dimensions was intended. The first situation (“refusal to work”) illustrates the perceived justice of the distribution of scarce resources (distributive justice), e.g., the teacher’s attention in the classroom. The second one (“sent out”) describes issues of respectful interactions between the student and teacher (interpersonal justice) (Peter et al., 2013). Both dimensions are highly relevant in the context of inclusive schools. However, further investigations showed that this distinction could not be confirmed empirically. As a result, we will refer to them as vignette “sent out” and “refusal to work” in the following chapters. Text blocks giving information on the interacting student were systematically varied. Again, reference is made to previous research findings on the attitudes of teachers toward inclusion pointing to teachers’ negative attitudes toward children with a SEN in domains of behavioral problems and, to a lesser extent, learning difficulties (Avramidis et al., 2000; Avramidis and Norwich, 2002; de Boer et al., 2011; Langner, 2015; Schwab and Seifert, 2015; Lübke et al., 2016; Ruberg and Porsch, 2017). Descriptions of these SEN and a neutral description were included as a manipulation in the text vignettes to investigate justice cognitions (see Table 1). The justice rating of each vignette was assessed *via* three items. The first items asked the participants to rate the interaction from their perspective. The other two items requested the participants to assess the justice of the interaction from the perspective of the interacting student, and his/her classmates. Answers were given on a five-point rating scale (1 = unfair to 5 = fair) with a neutral midpoint. The three items were then aggregated into a scale using the mean.

**TABLE 1:** Wording of vignettes and their varying characteristic “special educational need”.

	Manipulation “special educational need”			
	**Vignettes**	**SEN learning difficulties**	**No SEN**	**SEN behavioral problems**
	“Refusal to work” (distributive justice)	Today, a worksheet is to be completed silently. A child refuses to do so. The teacher briefly reminds him/her to work on his/her tasks. During the rest of the lesson the teacher turns to the questions of the other pupils. The child has a much slower comprehension and is overwhelmed faster than other children. In most subjects, he or she is two years or more behind the average learning level expected at this age.	Today, a worksheet is to be completed silently. A child refuses to do so. The teacher briefly reminds him/her to work on his/her tasks. During the rest of the lesson the teacher turns to the questions of the other pupils. The child generally behaves rather ordinary in class and performs according to his or her age group.	Today, a worksheet is to be completed silently. A child refuses to do so. The teacher briefly reminds him/her to work on his/her tasks. During the rest of the lesson the teacher turns to the questions of the other pupils. The child has great difficulty in restraining itself and following the lesson permanently. It often gets into conflict with the staff and classmates at the school.
	“Sent out” (interpersonal justice)	During the teacher’s talk, a child disturbs the lesson by repeated loud interjections. The teacher reminds him to be quiet. After a few minutes, the child starts to disturb the lesson again. After the child starts swearing, the teacher interrupts her talk and asks the child to wait outside the classroom for the rest of the lesson. The child has a much slower comprehension and is overwhelmed faster than other children. In most subjects, he or she is two years or more behind the average learning level expected at this age.	During the teacher’s talk, a child disturbs the lesson by repeated loud interjections. The teacher reminds him to be quiet. After a few minutes, the child starts to disturb the lesson again. After the child starts swearing, the teacher interrupts her talk and asks the child to wait outside the classroom for the rest of the lesson. The child generally behaves rather ordinary in class and performs according to his or her age group.	During the teacher’s talk, a child disturbs the lesson by repeated loud interjections. The teacher reminds him to be quiet. After a few minutes, the child starts to disturb the lesson again. After the child starts swearing, the teacher interrupts her talk and asks the child to wait outside the classroom for the rest of the lesson. The child has great difficulty in restraining itself and following the lesson permanently. It often gets into conflict with the staff and classmates at the school.

*Translated from German.*

The following instruments were only answered by the sample of in-service teachers. The items measuring attitudes toward inclusion were based on a questionnaire designed by Kopp (2009) (*M* = 2.51, *SD* = 0.67, Cronbach’s α = 0.80). Four items were rated on a four-point Likert scale. Experiences with persons with disabilities in personal and professional context were measured using 14 items (Forlin et al., 2010; Kim, 2011). The participants indicated for each item whether it applied to their experiences or not (multiple choice) (see Table 2). They also indicated which grades they taught. This was used to model the school type mainly taught in. The gender and age of the participants were collected as control variables.

**TABLE 2:** Descriptive statistics for the variables observed.

	Pre-service teachers	In-service teachers	Pre-service/in-service teachers
	***M (SD)***	***M (SD)***	***Cronbach’s* α**
Vignette “refusal to work”	2.71 (0.32)	2.43 (0.74)	0.60/0.68
Vignette “sent out”	3.18 (0.27)	2.88 (0.76)	0.42/0.61
	**example item**	***M (SD)***	***Cronbach’s* α**

Attitudes towards inclusion (based on Kopp, 2009)	“Inclusive teaching can meet the needs of all pupils through appropriate methods.”	2.51 (0.67)	0.80
	**example item**	**Applied to (in%)**	

Experiences with persons with disabilities in personal and professional context (based on Forlin et al., 2010; Kim, 2011)	“In my family are persons with disabilities.”	29.79	

*For wording of vignettes see Table 1. N (in-service teachers) = 2,254. N (pre-service teachers) = 275.*

### Design and Procedure

The in-service teachers rated the vignettes and items described above as a part of the project “Evaluation of inclusive schools in the state of Brandenburg, Germany” at the beginning of 2019. Following research question one, the descriptions of the student’s behavior pointing to a SEN were varied in the text vignettes as contextual information to investigate their influence on the teachers’ judgments of fairness in teaching situations. The description of a student with behavioral problems exclusively depicts the symptoms of an externalizing behavioral disorder (see Table 1), internal disorder patterns are not considered in the present design. The varying description of the student’s behavior was given at the end of each vignette. Accordingly, three variations of each of the two vignettes were developed. Three sets containing one variation of each vignette were generated. The participants were randomly assigned to one set, resulting in every participant seeing two of the three experimental variations in random order.

The pre-service teachers assessed these vignettes alongside four others during a lecture for pre-service teachers studying to teach at secondary level I and II at the University of Potsdam, Germany. The online survey was part of the ProfInk research program (Knigge et al., 2020). It was conducted using the online survey software “EFS Survey.” The design of this study is described in Kobs et al. (2021). It is important to note here that the information about the SEN of the interacting student was given at the beginning of each vignette for the pre-service teachers.

### Statistical Analyses

As every participant rated both vignettes, the data collected can be categorized as repeated measure. We analyzed the data using linear mixed effect models (LMM) (Singmann and Kellen, 2019). LMMs were computed with the lme4 (Bates et al., 2015b) and RePsychLing (Bates et al., 2015a) packages using the maximum-likelihood estimator and BOBYQA optimizer, and *p*-values were computed with the lmerTest (Kuznetsova et al., 2017) package, using Satterthwaite approximation for degrees of freedom. Effect sizes were computed with the effectsize package (Ben-Shachar et al., 2020). All statistical analyses were conducted using R (R Core Team, 2020).

To test for research question one, the experimental manipulation (description of SEN) was modeled in a within-subject three-level factor. To account for the difference of the two vignettes, a two-level factor was implemented. The control variable gender was also converted to a factor with two levels, the control variable age was centered around the mean (*M* = 49.18). In line with research question two, the attitudes toward the inclusion scale were added. For easier interpretability, it was centered around the mean (*M* = 2.51). To investigate research question three, the items relating to experiences with persons with disabilities were added. The 14 items were combined as follows to reduce model complexity. Knowing people with disabilities from activities in your free time and from your neighborhood was combined into the factor “acquaintance with disabilities.” Having a colleague with disabilities and having had a fellow student with disabilities were combined to the factor “colleague with disabilities.” Likewise, having had a student with disabilities during one’s own school years in their class or at their school was subsumed under the factor “persons with disabilities in own school days.” This resulted in nine two-level factors indicating whether the item(s) had applied to the participant or not. The type of school mainly taught in was modeled as a factor with three levels (primary school, secondary school, both equally).

To test for research question four, two models were computed. In the first model, pre-service and in-service teachers were compared. Since the participating pre-service teachers were studying to become secondary teachers, the sample of practicing teachers was reduced to secondary teachers for the comparison (*N* = 605). Again, the situational factor was modeled in a within-subject three-level factor, and a two-level factor accounted for the difference of the two vignettes. The difference in the participants’ professional status was modeled by a between-subject two-level factor. In the second model, only practicing secondary teachers with less than 15 years teaching experience were investigated (*N* = 198). This decision was based on literature implying a shift on attitudes toward inclusion for teachers with more than 10 years of teaching experience (see above). A three-level factor was implemented to account for up to 5, 10, and 15 years of teaching experience. Other model components were the same as in the previous model.

All factors were contrast coded using backward sliding contrasts (Venables and Ripley, 2002). This means that neighboring levels are compared, e.g., for the experimental variation (SEN) justice ratings for level 2 (no SEN) are compared to level 1 (learning difficulties), and level 3 (behavioral problems) is compared to level 2 (no SEN) (Schad et al., 2020). This is beneficial when multiple predictors are present, and interactions are modeled. Because of this coding, grand-mean centering is applied. Three models were computed to test for research questions one to three. The first model only included the control factors age and gender, the three-level factor of the manipulation and the factor representing the vignettes. Interactions of the latter two factors were computed as well. In the next model, we added the scale for attitudes toward inclusion and its interaction with the factor SEN. In the final model, 11 contrasts of the remaining factors (experiences with persons with disabilities and mainly taught school type) were entered. Interactions of the school type mainly taught in and the experimental variation were computed as well. All models included the participant’s ID as random effect. The effect sizes obtained from the effect size package were calculated using the test statistic to account for the dependency of the data. Accordingly, the ω^2^ obtained should be understood as an estimate (Ben-Shachar et al., 2020).

## Results

The teachers rated the vignette illustrating a more distributive situation (“refusal to work”) overall less just than the interpersonal vignette (“sent out”) (*M* = 2.43 vs. 2.88) (see Table 2). The internal consistency of both vignettes was acceptable with Cronbach’s α being 0.68 for the vignette “refusal to work” and 0.61 for the vignette “sent out.”

As described above, three models were computed to analyze the possible impact of situational and individual factors on the justice judgments of teachers. The first model contained only the experimental variations and vignette factor as well as the age and sex of the in-service teachers. The model’s total explanatory power was substantial (conditional *R*^2^ = 0.41), and the part related to the fixed effects alone (marginal *R*^2^) was 0.19. With adding the centered attitudes of inclusion scale, the explanatory power related to the fixed effects increased (marginal *R*^2^ = 0.21). A further addition of the items concerned with experiences with persons with disabilities and professional experiences led to a further increase of marginal *R*^2^ (0.23). The Akaike Information Criterion decreased from model 1 through model 3. Therefore, the following descriptions refer to model 3.

To answer research question four, two more models were computed to investigate the influence of teaching experience on justice judgment. We modeled increasing professional development by computing a factor illustrating completed training/education. As stated above, only secondary in-service and pre-service teachers were included in this analysis. The model’s total explanatory power was substantial (conditional *R*^2^ = 0.43), and the part related to fixed effects alone (marginal *R*^2^) was 0.18. The model, analyzing the influence of increasing teaching experience among in-service secondary teachers, explained *R*^2^ = 0.47 in total and the part related to fixed effects accounted for *R*^2^ = 0.21.

### Hypothesis 1: Influence of the Contextual Factor ‘SEN’ on Justice Ratings of Teachers

**FIGURE 1:**
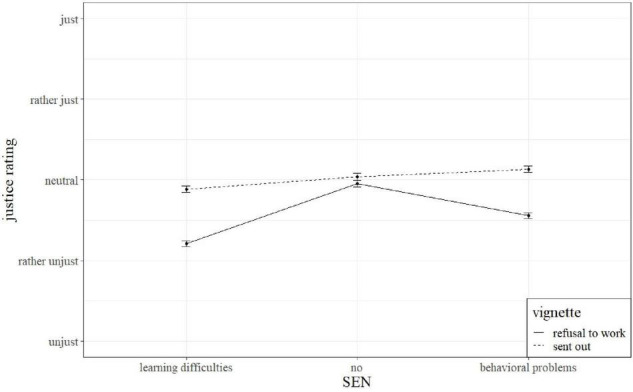
Interaction of vignette and special educational need (SEN).

The results obtained from the analyses described above are presented in Table 3. The results indicate that the behavioral descriptions did alter the justice ratings of the teachers. As shown in Table 3, the justice scores decreased by half a unit if the student’s behavior was described according to learning difficulties compared to the neutral description (*M* = 2.38 vs. 2.88, *p* < 0.001, ω^2^ = 0.04). Similarly, the teachers rated the text vignettes more just if the behavior of the student was described in a neutral way compared to a description of a student with behavioral difficulties (*M* = 2.88 vs. 2.70, *p* < 0.001, ω^2^ = 0.00). Both significant contrasts of the manipulation were of small effect size (Funder and Ozer, 2019). Since research in this field is scarce, an adequate classification is not yet possible. Moreover, in an experimental research setting, smaller effects are to be expected, especially when very complex designs are applied (Funder and Ozer, 2019). The significant interactions of the vignette factor and the experimental variations indicated a varying effect of the student’s behavioral description on justice ratings. Both effects were of small magnitude (Funder and Ozer, 2019). Figure 1 illustrates this. The teachers rated the situation “refusal to work” less just if the student was described with a special need (solid line). However, for the vignette “sent out” this is not true. The interaction was rated least just for the student with learning difficulties, and it was rated more just when the interacting student is described with behavioral difficulties, with the rating of the situation with a student without a SEN in between (dashed line).

**TABLE 3:** Fixed effects for mixed models predicting justice ratings of in-service teachers (*N* = 2,254).

	Unstandardized estimate b [95% CI]									

	**M1**			**M2**			**M3**			
***Parameter***	***Estimates***	***SE***	***t***	***Estimates***	***SE***	***t***	***Estimates***	***SE***	***t***	***Partial*ω*^2^***
(Intercept)	2.81***	0.06	48.61	2.85***	0.06	50.22	2.80***	0.06	46.02	
	[2.70, 2.92]			[2.73, 2.96]			[2.68, 2.92]			
**Experimental/**										
**situational factor**										
No SEN – learning difficulties (ld)	0.50***	0.02	21.07	0.50***	0.02	21.31	0.45***	0.04	11.76	0.04 [0.03,0.05]
	[0.46, 0.55]			[0.46, 0.55]			[0.37, 0.52]			
Behavioral problems – no SEN (bp)	–0.17***	0.02	–7.01	–0.17***	0.02	–6.99	–0.15***	0.04	–3.98	0.00 [0.00,0.01]
	[–0.21, –0.12]			[–0.21, –0.12]			[–0.22, –0.08]			
**Control factors and interactions**										
Vignettes “sent out” – “refusal to work” (vig)	0.45***	0.02	24.93	0.45***	0.02	24.92	0.45***	0.02	24.98	0.22 [0.19,0.25]
	[0.41, 0.48]			[0.41, 0.48]			[0.41, 0.48]			
Sex (male – female)	0.15***	0.03	4.69	0.14***	0.03	4.36	0.12***	0.03	3.60	0.01 [0.00,0.01]
	[0.09, 0.22]			[0.08, 0.20]			[0.05, 0.18]			
Age (centered)	–0.00	0	–1.83	–0.00**	0	–2.62	–0.00**	0	–2.71	0.00
	[–0.00, 0.00]			[–0.01, –0.00]			[–0.01, –0.00]			[0.00, –0.01]
ld × vig	–0.60***	0.05	–10.89	–0.59***	0.05	–10.88	–0.59***	0.05	–11.16	0.04 [0.03,0.05]
	[–0.71, –0.49]			[–0.69, –0.48]			[–0.70, –0.49]			
bp x vig	0.49***	0.05	8.99	0.49***	0.05	9.04	0.49***	0.05	9.24	0.03 [0.02,0.04]
	[0.39, 0.60]			[0.38, 0.59]			[0.39, 0.59]			
**Individual factors and interactions**										
Attitudes toward inclusion (centered) (ati)				–0.17***	0.02	–10.03	–0.16***	0.02	–8.94	0.03 [0.02,0.05]
				[–0.21, –0.14]			[–0.19, –0.12]			
ld x ati				0.09*	0.03	2.49	0.08*	0.03	2.33	0.00 [0.00,0.01]
				[0.02, 0.15]			[0.01, 0.15]			
bp x ati				–0.01	0.03	–0.42	–0.02	0.03	–0.48	0.00 [0.00,0.00]
				[–0.08, 0.05]			[–0.08, 0.05]			
Family member with disabilities (yes – no)							–0.07**	0.03	–2.81	0.00 [0.00,0.01]
							[–0.12, –0.02]			
Acquaintance with disabilities (yes – no)							–0.03	0.02	–1.29	0.00 [0.00,0.00]
							[–0.08, 0.02]			
Colleagues with disabilities (yes – no)							0.03	0.03	1	0.00 [0.00,0.00]
							[–0.02, 0.07]			
Persons with disabilities in own school days							–0.05	0.03	–1.86	0.00 [0.00,0.00]
(yes – no)							[–0.10, 0.00]			
Teaching experience in primary/secondary school with persons with disabilities (yes – no)							–0.01	0.04	–0.18	0.00 [0.00,0.00]
							[–0.08, 0.06]			
Teaching in special needs schools (yes – no)							–0.07*	0.03	–2.18	0.00 [0.00,0.01]
							[–0.14, –0.01]			
No teaching experience with persons with disabilities (yes – no)							0.02	0.04	0.57	0.00 [0.00,0.00]
							[–0.06, 0.11]			
Observing colleague teaching persons with disabilities (yes – no)							–0.07*	0.03	–2.35	0.00 [0.00,0.01]
							[–0.12, –0.01]			
Experiences with caretaking of persons with disabilities outside of school (yes – no)							–0.08*	0.04	–2.14	0.00 [0.00,0.01]
							[–0.15, –0.01]			
School type							0.12***	0.03	4.82	0.01 [0.00,0.02]
Secondary school – primary school (sec)							[0.07, 0.17]			
School type both – secondary school (bo)							–0.03	0.05	–0.48	00 [0.00,0.00]
							[–0.13, 0.08]			
ld × sec							–0.01	0.05	–0.29	00 [0.00,0.01]
							[–0.11, 0.08]			
bp × sec							–0.11*	0.05	–2.22	00 [0.00,0.00]
							[–0.21, –0.01]			
ld × bo							–0.18	0.11	–1.67	00 [0.00,0.00]
							[–0.40, 0.03]			
bp × bo							0.19	0.11	1.72	00 [0.00,0.00]
							[–0.03, 0.40]			
**Model information**										
AIC	9482.27			9383.06			9331.42			
ICC	0.28			0.26			0.24			
Observations	4,505			4,505			4,505			
Marginal *R*^2^/Conditional *R*^2^	0.186/0.411			0.209/0.413			0.226/0.415			

CI, confidence interval; ld, contrast no SEN – SEN learning difficulties; bp, contrast SEN behavioral problems – no SEN; vig, contrast vignettes “sent out” – “refusal to work”; ati, centered attitudes toward inclusion scale; sec, school type secondary school – primary school; bo, both school types – secondary school. p-values based on Satterthwaite estimation. *p < 0.05, **p < 0.01, ***p < 0.001.

### Hypotheses 2: Link Between Teacher’s Attitudes Toward Inclusion and Justice Ratings

**FIGURE 2:**
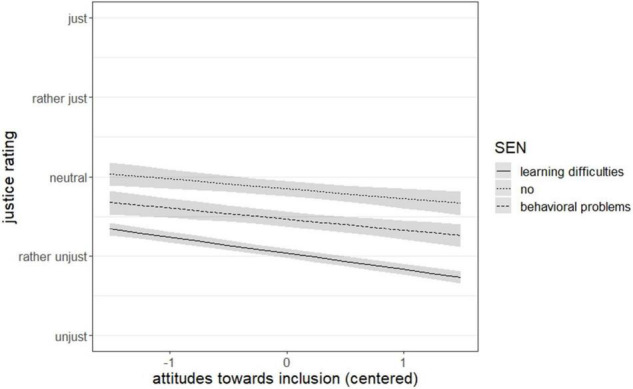
Interaction of attitudes toward inclusion and special educational need.

The results indicate a connection between teacher’s attitudes toward inclusion and justice judgments. An increase in attitudes toward inclusion can be associated with a decrease by 0.15 units on the scale of justice ratings (*p* < 0.001, ω^2^ = 0.03) (see Table 3). Again, this was an effect of small size (Funder and Ozer, 2019). There was also a significant interaction of attitudes toward inclusion and the experimental variation. Figure 2 illustrates this relationship: With an increase in attitudes toward inclusion, the decrease of the justice scores is stronger for situations in which the student is described with learning difficulties (dashed line) compared to a student without SEN (dotted line) (*p* < 0.05). The justice ratings were not significantly different for the SEN behavioral problems.

### Hypotheses 3: Influence of Individual Factors Concerned With Experience With Persons With Disabilities on Justice Ratings of Teachers

**FIGURE 3:**
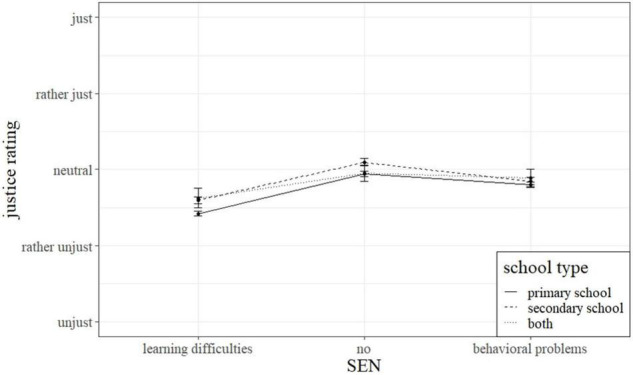
Interaction of school type mainly taught in and special educational need.

Six of the entered 15 contrasts were significant. The results suggest that subjectively experienced justice is related to personal and professional experiences of teachers with people with disabilities. Thus, teachers with family members with disabilities assessed the situations presented less fairly than their colleagues (*M* = 2.59 vs. 2.68, *p* < 0.01, see Table 3). Having taught children in special needs schools also negatively affected the justice ratings (*M* = 2.53 vs. 2.68, *p* < 0.05). Furthermore, having sat in on a colleague teaching an inclusive class also negatively influenced the justice ratings of the described interactions (*M* = 2.57 vs. 2.68, *p* < 0.05). Teachers who reported to have taken care of children outside of school also rated the described interactions lower than colleagues who did not (*M* = 2.53 vs. 2.67, *p* < 0.05). In addition, we found that secondary school teachers assessed the hypothetical descriptions generally fairer than primary school teachers do (*M* = 2.75 vs. 2.60, *p* < 0.001) (see also Figure 3). Lastly, there also was a significant interaction of the school type a teacher taught in and the experimental variation. Figure 3 illustrates this interaction. Primary and secondary school teachers rated both situations rather similar if a student is described with behavioral problems (*M* = 2.68 vs. 2.75). However, the secondary school teachers’ assessment of the situations increased steeper than that of the primary school teachers if the student is described neutrally (*M* = 3.01 vs. 2.82).

### Hypotheses 4: Influence of Individual Factors Concerned With Teaching Experience on Justice Ratings of Teachers

**FIGURE 4:**
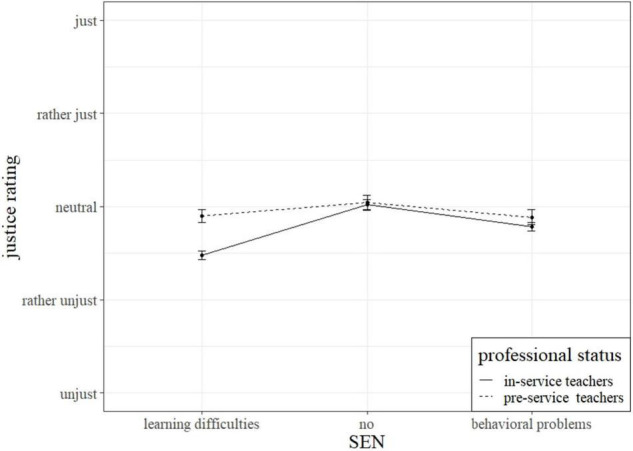
Interaction of teaching experience and experimental manipulation.

The results obtained from the analyses described above are presented in Table 4. The left column (M4) represents the results comparing pre-service and in-service teachers, the right column shows the results of the model estimating the influence of increasing teaching experience on justice ratings (M5). The analysis showed that pre-service and in-service teachers differed significantly in their justice ratings. The secondary in-service teachers rated both situations significantly lower than pre-service teachers (*M* = 2.76 vs. 2.94). Furthermore, the experimental variation and professional status interacted significantly. The significant interaction of the training status and the experimental variations indicates a varying effect of the student’s behavioral description in relation to the teaching experience of the person rating these descriptions. Figure 4 illustrates how the pre-service teachers generally rated the described interactions more just than practicing teachers. Describing the interacting student with learning difficulties enhances the different assessment. However, focusing solely on practicing secondary teachers no significant effect of teaching experience could be found.

**TABLE 4:** Fixed effects for mixed models predicting justice ratings for pre-service teachers and in-service teachers at secondary level in comparison (M4) and secondary teachers only (M5).

	Unstandardized estimate b [95% CI]					

	**M4**			**M5**		
***Parameters***	***Estimates***	***SE***	***t***	***Estimates***	***SE***	***t***
(Intercept)	2.85***	0.02	139.16	2.74***	0.04	63.44
	[2.81, 2.89]			[2.65, 2.82]		
**Experimental/situational factor**						
No SEN – learning difficulties (ld)	0.34***	0.04	8.57	0.51***	0.08	6.24
	[0.27, 0.42]			[0.35, 0.66]		
Behavioral problems – no SEN (bp)	–0.20***	0.04	–5.00	–0.25**	0.08	–3.12
	[–0.27, –0.12]			[–0.41, –0.09]		
**Control factors and interactions**						
Vignettes “sent out” – “refusal to work” (vig)	0.47***	0.03	17.12	0.52***	0.06	8.70
	[0.42, 0.53]			[0.40, 0.63]		
vig × ld	–0.55***	0.09	–6.43	–0.72***	0.19	–3.77
	[–0.72, –0.38]			[–1.10, –0.35]		
vig × bp	0.28**	0.09	3.22	0.56**	0.19	2.88
	[0.11, 0.45]			[0.18, 0.93]		
**Individual factors and interactions**						
Professional status	0.18***	0.04	4.45			
Pre-service t. – in-service t. (prof)	[0.10, 0.26]					
prof × ld	–0.39***	0.08	–4.88			
	[–0.55, –0.23]					
prof × bp	0.08	0.08	0.97			
	[–0.08, 0.23]					
Teaching experience				0.04	0.10	0.46
Up to 10 – up to 5 years				[–0.14, 0.23]		
Teaching experience				–0.07	0.11	–0.63
Up to 15 – up to 10 years				[–0.28, 0.14]		
**Model information**						
AIC	3661.648			858.264		
ICC	0.31			0.33		
*N*	880 _subj_			198 _subj_		
Observations	1,760			396		
Marginal *R*^2^/Conditional *R*^2^	0.185/0.434			0.207/0.466		

CI, confidence interval; ld, contrast no SEN – SEN learning difficulties; bp, contrast SEN behavioral problems – no SEN; vig, contrast vignettes “sent out” – “refusal to work”; prof, contrast professional status preservice teachers – in-service teachers. p-values based on Satterthwaite estimation. *p < 0.05, **p < 0.01, ***p < 0.001.

### Additional Results

Throughout all models the assessments of the two situations differed significantly, with the situation “sent out” being rated more just (see Tables 3, 4). Surprisingly, the results also suggest gender differences in evaluating the described interactions with male teachers assessing them generally more just than females (*M* = 2.79 vs. 2.63). Since 84% of the sample were female, this finding should be taken with caution.

## Discussion

This study investigated whether context and individual factors influence teacher’s justice ratings of hypothetical student-teacher-interactions. To do so, we used two text vignettes illustrating classroom interactions and experimentally varied the description of the interacting student to represent learning difficulties, behavioral problems, or no special needs (situational factor). These were then rated by our participants. Given the inclusive setting of the described interactions, we assessed the participants’ attitudes toward inclusion, experiences with persons with disabilities, and years of teaching experience (individual factors).

In line with hypothesis 1, we found a general effect of the experimental manipulation on teachers’ justice ratings since they rated both hypothetical interactions less just if the interacting student was described with a SEN. Equal treatment of all students in the social reference group (classroom) was rated less just if the interacting student was described with a SEN. This result is in line with the principle of need. Accordingly, a needs-based distribution of attention and appreciation might be preferable in the presence of SEN, even though this was not explicitly investigated in the present study. The reported general effect of SEN is partly in line with results of Kobs et al. (2021) who found a significant, overall effect only for the manipulation “behavioral problems” on justice ratings of student teachers.

Closer inspection revealed significant interactions of the text vignettes and the experimental manipulation. The justice ratings decreased for the vignette “refusal to work” in the presence of SEN. Again, a preference for a needs-based distribution of attention instead of equal treatment in case of SEN could explain this rating trend and is in line with the findings of Kobs et al. (2021) and consistent with the findings of Berti et al. (2010) who reported teachers’ preferences for needs-based distribution of resources. When it comes to distributing resources in the learning environment, teachers seem to reference the principle of need. For the interaction described in “sent out” this pattern did partly replicate. Consistent with hypothesis 1, the justice rating of this text vignette was rated less just if a student was described with learning difficulties compared to a student with a neutral description. Again, a preference for the principle of need seemed to guide these ratings. However, sending out a student with behavioral problems was rated more just than the same interaction with a student without SEN (see Figure 1). Teachers might have rated the interaction for this SEN more just because they prioritize having a calm learning environment for the rest of the class and therefore felt that this action was justified. The principle of need does not seem to guide this rating instead a preference for equal treatment in this situation could explain this rating trend. This outcome is contradictory to that of Kobs et al. (2021) who reported opposite pre-service teacher ratings for this vignette. Overall, these findings indicate that our participants considered an equal distribution of resources in the described classroom situations to be less fair if a student with a SEN was present. This was true for both SEN. It supports our assumptions that the need principle is relevant in justice cognitions in the context of inclusive classroom settings (Peter et al., 2013). The consistent application of the needs principle in assessing the fairness of both situations with a student with learning difficulties also points to our participants’ knowledge on adaptive teaching strategies for this SEN (Lübke et al., 2016). Regarding students with behavioral problems, the teachers in our study seemed to distinguish in their assessment depending on the interaction described. As stated above, Ehrhardt-Madapathi et al. (2018) found a negative relationship between teachers’ justice perceptions of their own actions and students’ behavioral problems, thus not paying attention to a child with behavioral problems might make more sense in certain contexts to avoid enhancing the problem. This highlights the importance of context when it comes to justice. Since the situational descriptions were kept very short, the teachers might have varying assumptions with regard to relationship dynamics of the vignette protagonists or other interactions preceding this situation. For these reasons, hypothesis 1 could only be partly confirmed.

The extent to which individual abilities of perspective-taking and empathy can play a role here and contribute to a further expansion of the acceptance and use of the need principle appears to be of great relevance for future studies to gain a deeper understanding of the evaluation processes that take place here. Due to the experimental design, this aspect was kept randomly constant in the present study. How justice-sensitive a person is in general (Baumert and Schmitt, 2016) and possible links between the facets of justice sensitivity and the perspectives taken here while rating the student–teacher-interactions are other aspects to consider in future research. For example, rating the text vignettes from the student’s perspective could appeal to a large extent to the respondents’ observer sensitivity and the classmate perspective could appeal to the respondent’s observer or maybe even beneficiary sensitivity. Thus, investigating possible relationships between facets of injustice sensitivity and individual perspectives in the items could be promising. A corresponding emphasis on strengthening the different perspectives on justice would seem worthwhile for further studies. As stated above, the brevity of the text vignettes might have encouraged our participants to speculate about previous interactions of the vignette protagonists and their relationship dynamics. This could lead to different assumptions that influence their justice ratings. To gain a deeper understanding of justice cognitions in educational settings teachers’ reasoning and their understanding of the presented situations could be explored in think-aloud protocols in future studies (e.g., Paseka and Hinzke, 2014).

As described above, we hypothesized a link between attitudes toward inclusion and justice ratings of inclusive teaching interactions. The justice ratings of the text vignettes generally decreased with more positive attitudes toward inclusion of the teachers. Several studies suggest a positive link between knowledge about inclusion or SEN and attitudes toward inclusion (Avramidis and Norwich, 2002; de Boer et al., 2011), which might explain the decreasing justice ratings in our study if the principle of need is not applied in a situation with a student with SEN present. Although this is an effect of small size, adding attitudes toward inclusion and interactions with the experimental variation to our model led to an increase of 2% in variance explained (marginal *R*^2^). These findings support our hypothesis that more positive attitudes toward inclusion could be linked to a heightened awareness of students’ individual needs. A significant interaction was also found for attitudes toward inclusion and the experimental variation. The justice ratings of the vignettes decreased more strongly as attitudes toward inclusion increased when the student was described with learning difficulties compared to a neutral student description. This effect was not found for the description of a student with behavioral problems. This differentiation in relation to the specific SEN might be explained by varying attitudes for these SEN (Lübke et al., 2016). Still, these findings support our claim, that more positive attitudes toward inclusion can be associated with a heightened awareness for individual needs of children with SEN resulting in a preference for the need principle in justice ratings of these vignettes.

Regarding hypothesis 3, the results are not pointing in a clear direction. Some of the experiences the participants had seem to be linked to justice ratings. A link between experiences with persons with disabilities and justice cognitions has so far not been investigated. Following hypothesis 2 and adopting existing research about a possible relationship of these experiences and attitudes toward inclusion, might help explain some of these findings. In their review on attitudes toward inclusion, de Boer et al. (2011) report a positive link between experiences with persons with disabilities outside of a professional context and attitudes toward inclusion. This might also relate to the negative influence having a disabled family member had on justice ratings. Still, being acquainted with or having had a classmate with disabilities did not influence justice ratings. Some experiences with persons with disabilities in a professional context can be linked to more critical justice ratings, namely teaching experience in special needs schools, having observed one’s colleagues teaching in a diverse classroom, and taking care of persons with disabilities outside of school. Again, literature about attitudes toward inclusion helps to understand these findings since a positive effect of professional contact to persons with disabilities could be found (de Boer et al., 2011; Ruberg and Porsch, 2017). Thus, rating these interactions less just could be due to more experiences and in turn a heightened awareness for the needs of students with SEN. However, several other factors regarding private and professional experiences with persons with disabilities did not seem to affect the teachers’ justice ratings. All in all, these findings do not follow a clear pattern. Besides the self-reported experiences with person with disabilities, primary school teachers consistently rated the text vignettes lower as their colleagues teaching secondary students. The further development of inclusive education in primary schools and the corresponding longer experience with the needs of children with SEN could be a reason for this (Avramidis and Norwich, 2002; de Boer et al., 2011; Hellmich and Görel, 2014). Another reason might be, that needs-based instruction is more appropriate at the primary level than at the secondary level, where the focus is on achievement. Accordingly, a violation of the needs principle in the vignettes provokes more negative justice ratings among primary teachers than among secondary teachers (Ehrhardt et al., 2016). In conclusion, it remains unclear whether personal and professional experiences with persons with disabilities can be linked to a heightened awareness for the individual needs of children with SEN in terms of justice. In this study, a clear pattern that supports this claim could not be found.

In line with hypothesis 4, pre-service teachers were less critical of the described classroom interactions and rated them generally more just than practicing teachers. This difference could be explained by practicing teachers’ enhanced ability to notice and interpret situational factors relevant to teaching in general, due to their teaching experience (König et al., 2014; Paseka and Hinzke, 2014; Voss et al., 2015). Their extensive knowledge about diverse learners in theory and practice could have led to them more strongly preferring a needs-based justice rating of the described interactions. We further inspected an influence of teaching experience by focusing on in-service secondary teachers in their first 15 years of teaching. This was again inspired by research on attitudes toward inclusion (Avramidis and Norwich, 2002; de Boer et al., 2011). However, we found no effect of teaching experience in the first 15 years on justice ratings. Following hypothesis 2, this was surprising since teachers with less teaching experience were found to have more positive attitudes toward inclusion in the literature (Avramidis and Norwich, 2002; de Boer et al., 2011) which should have led to higher justice ratings with increasing teaching experience. Therefore, hypothesis 4 could only partly be verified.

### Limitations

The generalizability of these results is subject to certain limitations. For instance, the effects found in line with hypothesis 1 are partly limited by the design of our instrument. In the investigated interactions, the student in focus is treated the same as his classmates (principle of equality). Whether an increase in attention from the teacher would also be judged as fairer is unclear. According to the literature, a negative deviation from equal treatment is potentially more likely to cause perceived injustice than a positive deviation (Gollwitzer and van Prooijen, 2016). Therefore, a positive deviation from equal treatment of all students could lead to other effects, especially in inclusive education and in terms of the need principle. Further investigating the effects of SEN with a positive deviation from the principle of equality is in preparation.

Regarding hypothesis 4, the reported effects are limited by the different specializations of the two samples we compared. A part of the pre-service teachers studying to teach in secondary schools is specializing to teach in Grammar Schools which are usually less inclusive (forsa Politik- und Sozialforschung GmbH, 2017; Allan and Sturm, 2018). On the contrary, all of the in-service secondary school teachers worked in comprehensive secondary schools with a focus on inclusive education. The described effects could, therefore, be due to this difference in specialization. Nonetheless, an enhanced ability of in-service teachers to notice and interpret situational factors relevant to teaching regardless of their specialization can be assumed; still, further research with Grammar School teachers is needed here.

Another limitation is the subject-wise unbalanced manipulation, since the participants rated two of the three variations of the manipulation. LMMs generally cope well with unbalanced designs and yield plausible results (Singmann and Kellen, 2019). Nonetheless, the number of ratings were evenly distributed across each manipulation and vignette (around 700 per cell), so the subject-wise imbalance is subordinate.

It is unfortunate that we could not include more vignettes in the study. A higher number of vignettes would have been useful to better account for the variance of the individual vignettes in the model. Following the recommendations, 10–20 vignettes could have yielded plausible estimates as random effects in our model (Bates et al., 2015a; Singmann and Kellen, 2019). Nevertheless, to control for the differences of the two situations at least partially in the present study, we modeled them as a fixed effect. This allowed for a more accurate estimate of the effects of the implemented manipulation. A higher number of vignettes places significant demands on a study design. However, feasibility should be thoroughly examined in future studies.

### Implications and Prospects for Future Research

This study has shown that in-service teachers are aware of justice issues in inclusive teaching settings. Due to its experimental approach, it is unclear whether these findings can be generalized to real life teaching situations. However, other vignette studies have shown that the obtained results can be transferred to real life actions (Eifler, 2008; König et al., 2014). Consequently, a transfer to real classroom situations is very likely. It seems important here to acknowledge that the instrument we used simplifies the complex reality of student-teacher interactions. Even with teachers’ best intentions to act justly in the classroom, feelings of injustice can arise in students. Interactions in the classroom are very complex and their perceptions are influenced by various factors, such as previous experiences. How students perceive similar situations and assess their fairness is another important research objective to learn more about justice cognitions of different participants of everyday school life.

The results presented here also indicate a link between attitudes toward inclusion and awareness for justice in inclusive teaching interactions. Whether teacher training on inclusion, possibly mediated by attitudes toward inclusion, would influence teachers’ justice awareness in inclusive teaching settings is a further research question that arises from this study. Similarly, how experiences with persons with disabilities might be connected to justice ratings needs to be further explored.

Following the reported link between attitudes toward inclusion and justice ratings in this study and findings on the effect of positive attitudes toward inclusion and its impact on student outcomes (Savage and Erten, 2015), investigating whether teachers’ awareness for justice in interactions on a school-level can be linked to student outcomes appears to be a promising objective for further studies.

## Data Availability Statement

The raw data supporting the conclusions of this article will be made available by the authors, without undue reservation.

## Author Contributions

SK wrote the first draft of the manuscript, designed the study, analyzed the data, and revised the manuscript. MK co-designed the study and contributed substantially to the manuscript. MK, AE, JL, AH, and NS designed and coordinated the project of which this study is a part, reviewed and edited the manuscript. All authors read and approved the final manuscript for submission.

## Conflict of Interest

The authors declare that the research was conducted in the absence of any commercial or financial relationships that could be construed as a potential conflict of interest.

## Publisher’s Note

All claims expressed in this article are solely those of the authors and do not necessarily represent those of their affiliated organizations, or those of the publisher, the editors and the reviewers. Any product that may be evaluated in this article, or claim that may be made by its manufacturer, is not guaranteed or endorsed by the publisher.

## Footnotes

1 Translated from German (Evaluation Gemeinsames Lernen und Schulzentren im Land Brandenburg).

2 Translated from German [Professionalisierung von (angehenden) Lehrkräften im Bereich Inklusion].
